# Expanding access to addictions care: Implementation of a 24-hour healthcare provider support line in British Columbia, Canada

**DOI:** 10.1186/s13722-024-00508-z

**Published:** 2024-10-31

**Authors:** Anjali Sergeant, Amanda Giesler, Nirupa Goel, Paxton Bach

**Affiliations:** 1 Department of Medicine, University of British Columbia, Vancouver, Canada; 2 British Columbia Centre on Substance Use (BCCSU), 1045 Howe Street, Suite 400, Vancouver, BC V6Z 2A9 Canada

## Abstract

**Background:**

Morbidity and mortality related to substance use have risen to catastrophic levels in North America, and treatment services are often difficult to access. In response, the province of British Columbia (BC), Canada, launched a province-wide addiction medicine support phone line that offers clinicians immediate access to phone consultation with an addictions medicine expert. The service operates 24/7 is accessible to any clinician in the province seeking assistance with an addiction-related question. We describe an evaluation of the reach and perceived impact of the service over its first two years.

**Methods:**

The 24/7 Addiction Medicine Clinician Support Line was evaluated prospectively from June 2020 to April 2022. All provider-to-provider encounters were included. Data was collected from two primary sources: health provider demographic information collected at the time of consultation, and optional clinician surveys conducted after the consultation was complete. Descriptive data are presented as numerical values and percentages.

**Results:**

Over the 22-month evaluation period, 1,279 consultations were requested by 631 distinct care providers across British Columbia. The service averaged 15 calls per week across the province, and 51.5% of calls were made outside of business hours. Physicians made the majority of calls to the service (*n* = 865, 67.6%), followed by nurse practitioners (*n* = 162, 12.7%). Among those who completed a follow-up survey (*n* = 258 calls, 20.2% total calls), 81.8% (*n* = 211) were “very” or “extremely” satisfied with the consultation. Of these respondents, 65.5% (*n* = 169) reported that the consultation led to the provision of better care for their patient, with 58.1% (*n* = 150) initiating a new prescription and 22.1% (*n* = 57) reporting expedited treatment for their patient. The consultation area of focus was most commonly opioid use (*n* = 417; 59.6%), followed by polysubstance use (*n* = 98; 14.0%).

**Conclusions:**

The impact of the 24/7 Addiction Clinician Support Line was widespread, and the service increased accessibility to evidence-based addictions treatment across a range of care settings. Clinicians expressed a high degree of satisfaction with the service. To our knowledge, this province-wide program is the first of its kind in North America, offering a scalable and adaptable model to support access to evidence-based addictions care in under-resourced settings.

**Supplementary Information:**

The online version contains supplementary material available at 10.1186/s13722-024-00508-z.

## Introduction

Morbidity and mortality related to substance use, particularly synthetic opioids, have risen to catastrophic levels in North America over the past decade [1, 2]. In British Columbia, unregulated drug overdose is now the leading cause of death among individuals aged 10 to 59, reflecting broader trends in drug-associated mortality across Canada and the United States [3, 4]. While evidence-based treatment options can reduce substance-associated harms, recent data suggests that only approximately 10% of people with substance use disorders access treatment [5]. Many primary and acute care providers feel ill-equipped to provide addictions care, a significant contributor to the treatment gap [6, 7]. The scarcity of addictions training among providers limits the appropriate prescription of potentially life-saving therapies for prevalent conditions such as opioid use disorder and alcohol use disorder [5, 8]. 

The treatment gap for people with substance use disorders exists in both outpatient and inpatient settings. Addiction consultation services can improve the quality of care among hospitalized patients with substance use disorders, but point-of-care addiction medicine services are often inaccessible in urgent care and inpatient settings [9]. Despite the known benefits of timely and evidence-based substance use disorder treatment, few addictions-focused provider support models are described in the literature [10]. In other medical specialties, telemedicine programs offering direct consultation to providers have resulted in improved treatment quality and patient outcomes [11]. Virtual medicine tools that support provider decision-making thus hold the potential to fill important gaps in addiction healthcare services, both in acute and outpatient care settings.

In response to persistent gaps in addictions care, the British Columbia Centre on Substance Use (BCCSU) launched the 24/7 Addiction Medicine Clinician Support Line in 2020. This service offers healthcare providers immediate consultation with an expert in addictions medicine. The service operates 24 h a day, 7 days a week, and is available to clinicians in the province of British Columbia (BC). This program is the largest of its kind in North America and the first to provide around-the-clock and point-of-care addiction medicine support to an entire state or province. We aim to evaluate this novel model of care and present findings on the reach and impact of the service.

## Methods

### Overview

The 24/7 Addiction Medicine Clinician Support Line was developed by the BCCSU alongside the British Columbia Ministry of Mental Health and Addictions and the Ministry of Health. Feedback from over 2,500 clinicians who participated in educational workshops held by the BCCSU was influential in the initiative’s development. Regardless of professional training or clinical setting, providers were keen to provide addictions care but desired additional support in clinical decision-making. Funding from the British Columbia provincial government supported the implementation and evaluation of this initiative. Initially, the BCCSU was supported with $500,000 Canadian dollars which was sufficient to run the service from 2020 to 2023. Due to its success, an additional budget to support the long-term continuation was granted by the province following the implementation and evaluation phase.

In order to operationalize a provincial provider consultation line, the BCCSU leveraged an existing provincial network of opioid agonist prescribers and addiction medicine specialists. After establishing clinical leadership for the service, approximately thirty physicians from across the province were recruited and trained to staff the line. Providers were selected based on their training in addiction medicine, past work experience, and current practice in order to ensure that they possessed an appropriately comprehensive level of expertise. The 24/7 addiction medicine consultation service was launched on June 16, 2020, with consultation services geared toward screening, assessment, treatment planning, and ongoing management for substance use and substance use disorders. The BCCSU worked in partnership with regional health authorities, the First Nations Health Authority, and clinician groups such as the BC Divisions of Family Practice and the Midwives Association of British Columbia to promote and raise awareness about the service. Information about the service was incorporated into all of the BCCSU’s professional training and educational curricula. The service’s outreach strategy involved a provincial Ministry of Health news release, the dissemination of service information to BC pharmacies and health authorities via fax and mail, and a promotional social media campaign.

Providers staffing the line received the patient’s personal health number and basic identification purposes to enable documentation of the consultation as a clinical encounter within a secure electronic medical record. However, patient-identifying information was not collected as part of the evaluation and quality control arm of the program, in an effort to protect patient and provider anonymity. Evaluation metrics for the consultation line involved basic provider demographic details and anonymized survey data from providers who called in to the line (see Methods: Data Collection below).

### Study design

**Fig. 1:**
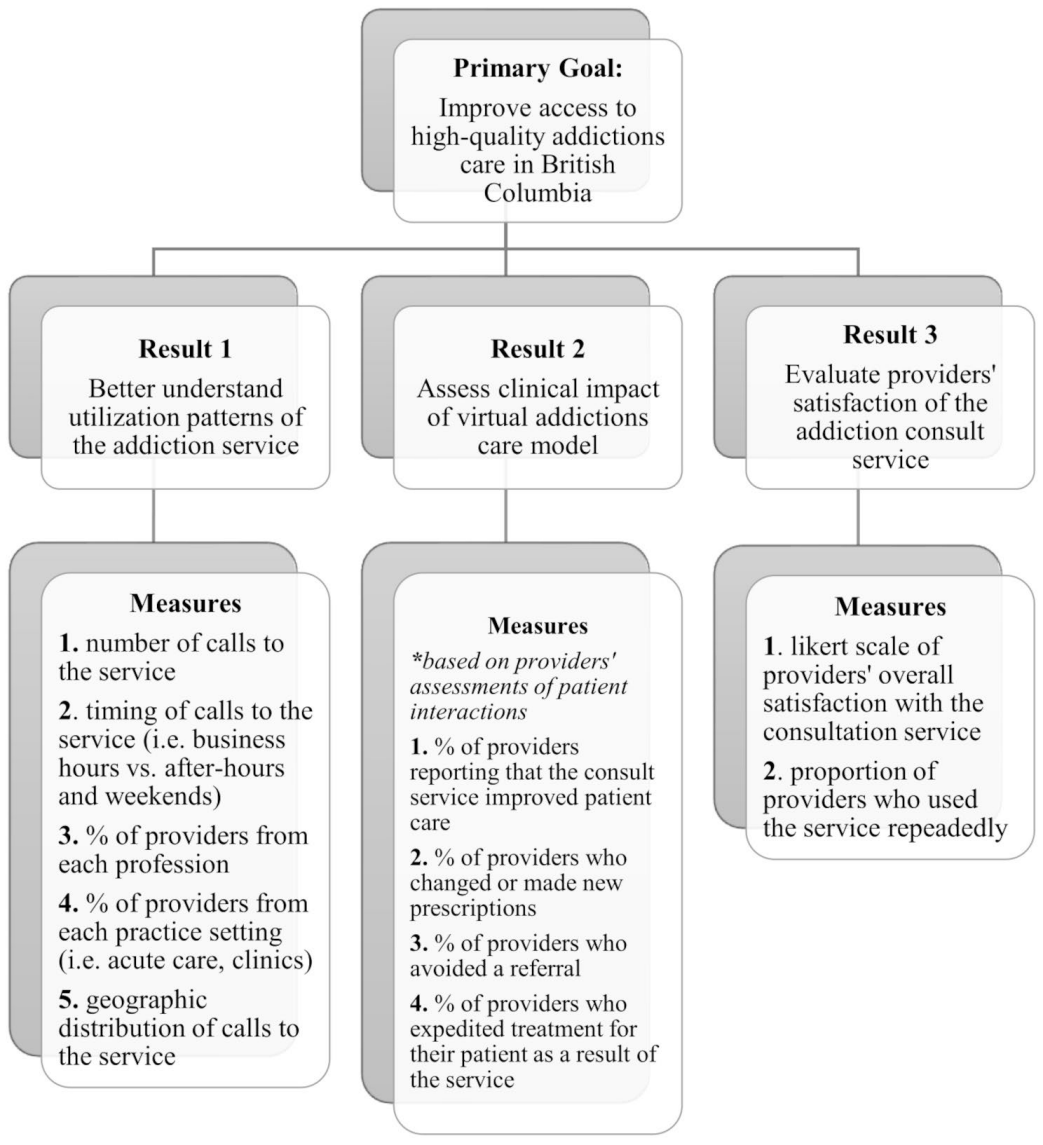
Illustrative results framework for the 24/7 addiction consult line

This study is a prospective, observational program evaluation of the 24/7 Addiction Medicine Clinician Support Line. We utilized a results framework in order to develop the study’s evaluation criteria with input from a multidisciplinary team of addictions experts, government health officials, and stakeholders in the province of British Columbia. The results framework has been applied to virtual health interventions in previous evaluation studies, and connects overall program objective(s) to intermediate results that are evaluated with clearly-defined performance measures [12]. With the primary goal of increasing accessibility to high-quality addictions care in British Columbia, the results focused on utilization patterns, clinical impact, and provider satisfaction (Fig. 1).

Given that this research was conducted as a clinical program evaluation, ethical approval was not required as per Articles 2.3 and 2.5 of the Tri-Council Policy Statement: Ethical Conduct for Research Involving Humans (TCPS-2) [13]. No identifiable patient or provider information was collected for research purposes and no data were linked.

### Participants

Any healthcare provider who called the 24/7 Addiction Medicine Clinician Support Line during the study period was included. The term ‘healthcare provider’ includes primary care physicians, specialist physicians, nurse practitioners, registered nurses, registered psychiatric nurses, pharmacists, and licensed midwives. Support line encounters that took place from the program’s inception in June 2020 until the completion of the study pilot phase in April 2022 were included.

### Data collection

Data were collected from two primary sources: (1) healthcare provider demographic information collected by the support line staff at each encounter and (2) optional surveys conducted through total population sampling of providers after the consultation took place. Overall frequency and timing of calls to the 24/7 Addiction Medicine Clinician Support Line were monitored throughout the study period. Clinicians consented to the collection of demographic and clinical information for research purposes, including healthcare profession, clinical setting, geographic location, and consultation type (by drug class). Data on the type of substance use focused on during the consultation was collected for the first 700 encounters to gain a sense of providers’ needs when seeking addictions expertise. Geographic region was categorized by health authority; the province’s health administration is divided into seven health authorities that direct regional care in British Columbia. Callers had the option to provide partial consent to the collection of certain demographic variables only (i.e. healthcare profession but not geographic location), or to opt out of data collection entirely. Following the encounter, providers were sent an optional survey over text message that asked questions about their satisfaction with the consultation and any modifications to patient care following the encounter (Supplement Table 1). A comprehensive list of study outcome measures is detailed in Fig. 1. All data were anonymized and de-identified from individual encounters and stored in an encrypted and password-protected document. No patient data was stored.

### Data analysis

Categorical data are presented as numerical values and percentages. Given the descriptive nature of the evaluation no statistical analyses were performed. All cases of missing data are reported.

## Results

**Fig. 2:**
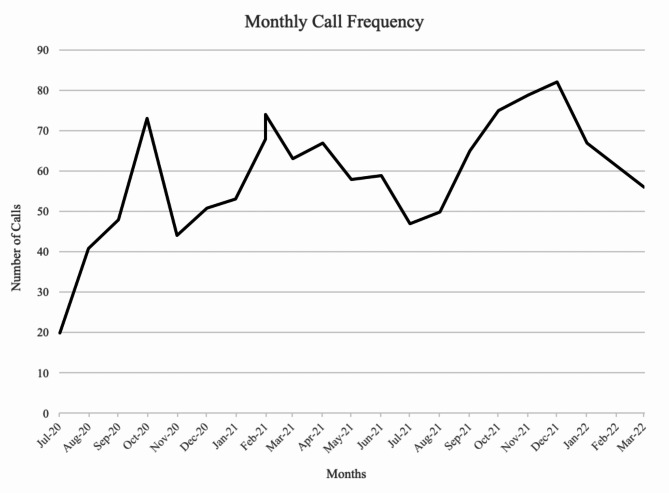
Monthly call frequency Monthly number of calls to the addiction consultation line from July 2020 to March 2022; June 2020 and April 2022 were excluded from the figure because calls were not collected for the entirety of the first and final months

Over the 22-month evaluation period, 1,279 consultations were requested by 631 unique care providers across British Columbia. There was an average of 59 monthly calls to the service (Fig. 2), with 51.5% of calls made outside of business hours (8:00 am to 5:00 pm). Physicians made the majority of calls to the service (*n* = 865, 67.6%), followed by nurse practitioners (*n* = 162, 12.7%), registered nurses (*n* = 113, 8.8%), and pharmacists (*n* = 106, 8.3%; Table 1). Practice settings were spread between community care clinics (*n* = 290, 22.7%), hospital emergency departments (*n* = 255, 19.9%), and inpatient or acute care settings (*n* = 271, 21.2%). Encounters originated from each of the provincial health regions, with the highest number originating from Fraser Health Authority (*n* = 302; 23.6%), followed by Vancouver Island Health Authority (*n* = 250, 19.5%) and Interior Health Authority (*n* = 158, 12.4%). Of the first 700 telephone consultations, the area of focus was most commonly opioid use (*n* = 417; 59.6%), followed by polysubstance use (*n* = 98; 14.0%) and alcohol use (*n* = 53; 7.6%).

**Table 1:** Baseline provider c﻿haracteristics

Call Characteristics total calls = 1279	*N* (%)*	Missing Data
Unique callers	631 (49.3)	9
Provider type		9
*Physician* *Nurse Practitioner* *Registered Nurses* *Pharmacists* *Other (i.e. midwives*,* social workers*,* care navigators)*	865 (67.6) 162 (12.7) 113 (8.8) 106 (8.3) 24 (1.9)	
Geographic location		321
*Vancouver Coastal Health Authority* *Fraser Health Authority* *Vancouver Island Health Authority* *Interior Health Authority* *Northern Health Authority* *Providence Health Authority* *First Nations Health Authority*	143 (11.2) 302 (23.6) 250 (19.5) 158 (12.4) 66 (5.2) 30 (2.3) 9 (0.7)	
Provider clinical setting		342
*Community-based care* *Emergency department* *Inpatient care* *Other/Unspecified*	290 (22.7) 255 (19.9) 271 (21.2) 121 (9.5)	
Substance use area of focus**		–
*Opioids* *Alcohol* *Benzodiazepines* *Stimulants* *Polysubstance use* *Other/Unspecified*	417 (59.6) 53 (7.6) 13 (1.9) 13 (1.9) 98 (14.0) 106 (15.1)	

*percentages may not add to 100% to due missing data

**substance use data was collected for the first 700 encounters of the line

Among clinicians who completed a follow-up survey after an encounter (*n* = 258, 20.2% of total calls), 81.8% (*n* = 211) of service users were “very” or “extremely” satisfied with the consultation, 16.7% (*n* = 43) reported that they were “somewhat satisfied”, and 1.5% (*n* = 4) rated the calls as “unsatisfactory” (Table 2). A total of 58.1% (*n* = 150) of responders initiated a new prescription after the call and 17.0% (*n* = 44) modified a previous prescription. Of all respondents, 65.5% (*n* = 169) reported that the consultation led to the provision of better care for their patient, with 22.1% (*n* = 57) responding that the service expedited treatment and 10.4% (*n* = 27) reporting that the consultation avoided a referral to the emergency department or a specialist physician.

**Table 2:** Provider survey results

Caller Satisfaction total calls = 258	*N* (%)
Overall satisfaction with the addictions consult service	
*Extremely satisfied* *Very satisfied* *Somewhat satisfied* *Unsatisfied*	81 (31.4) 130 (50.4) 43 (16.7) 4 (1.5)
Consult improved patient care	169 (65.5)
Consult expedited treatment	57 (22.1)
Consult avoided referral to specialist or emergency dept.	24 (10.4)
New prescription initiated	150 (58.1)
Prescription modified	44 (17.0)

## Discussion

This paper describes the implementation of a provincial on-demand addiction medicine support line, which has demonstrated a positive impact across a breadth of geographic regions and practice settings. We developed the 24/7 Addiction Medicine Clinician Support Line with input from providers across the province, which likely contributed to its wide reach as a support tool. The model of clinician-to-clinician mentorship for substance use disorders has been established in adolescent and adult patient populations, but historically these services have not been designed to offer immediate clinical advice to guide management while a patient is seeking care [14, 15]. To our knowledge, there are few existing community-accessible addictions programs that offer point-of-care support to clinicians. The Drug and Alcohol Clinical Advisory Service established in Australia offers a similar 24/7 addictions teleconsultation model to providers practicing across multiple Australian states [16]. In the United States, the Maryland Addiction Consultation Service offers a state-wide service focused on medication for opioid use disorders (MOUD) prescribing and the California Poison Control System piloted a 24/7 state-wide clinician support hotline for opioid use disorder in urgent care settings [17, 18]. These innovative programs have helped to fill gaps in addictions care. Within this landscape, the 24/7 Addiction Medicine Clinician Support Line is the first in North America to offer around-the-clock and point-of-care advice for any clinician queries around any substance use disorder to a large population.

The provider consultation service was unique in its ability to provide real-time advice, which allowed clinicians to answer questions and provide treatment options around substance use during a patient’s care visit. Point-of-care clinician resources support the provision of effective and high-quality care [19, 20], and may prevent treatment delays and unnecessary referrals [21]. As evidenced by a high proportion of after-hours calls, the service also allowed providers to access case-specific addictions expertise when no other consultative services were available. Provider queries around opioid use disorder and its management made up almost two-thirds of calls to the service within the initial pilot phase, which reflects the high degree of need for opioid use disorder treatment [2]. Many of the calls involved discussion around multiple drug classes, offering insights into the importance of addictions expertise on a range of overlapping substance use disorders. After its pilot project, the California Poison Control System opioid use disorder program similarly noted that there was need for addictions expertise beyond opioid use disorder alone [18]. The 24/7 Addiction Medicine Clinician Support Line was accessed by providers from a diversity of geographic regions across the province, demonstrating both its reach as well as the need that exists for this type of support across a breadth of urban, suburban, and rural contexts. This aligns with evidence that virtual consultation services may be especially useful in under-resourced or rural communities [11]. 

Our work adds to promising research suggesting that virtual clinician support tools are generally well-received and can heighten provider confidence in managing complex conditions [22]. Though the available body of evidence is limited by a low number of interventions reported and heterogeneity in evaluation measures [21, 22], clinician-to-clinician consultation services hold the potential to greatly benefit patients by better supporting their care. We found that the 24/7 Addiction Medicine Clinician Support Line often impacted providers’ self-reported approaches to addictions care. Providers frequently altered their pharmacologic management of substance use disorders as a direct result of the consultation. In some cases, the service prevented a need for referral to urgent or specialist services. Importantly, several providers opted to use the service multiple times. Providers who completed a follow-up survey after the phone consultation expressed a high degree of satisfaction with the service, with two-thirds noting that the service led to a direct improvement in patient care.

Indigenous peoples in Canada are disproportionately impacted by substance use, in the context of centuries of colonial tactics and government-sponsored policies that have led to significant trauma and health disparities among this population [23, 24]. Despite the wide reach of the provider consultation line across the province, it is notable that only nine calls were placed from the First Nations Health Authority (FNHA). The FNHA was established ‘by and for First Nations’ people in 2013, with the goal of centering healthcare decision-making and governance within distinct First Nations communities across BC [25, 26]. The low uptake within the FNHA may underestimate the service’s use within Indigenous populations, as some providers working with these communities may also be affiliated with other geographic health authorities. The consultation line may also have been limited by the presence of other addictions services within the FNHA, including a virtual daytime addictions clinic designed by the health authority. Evidence suggests that community-based and culturally-oriented interventions can better promote Indigenous health, particularly when guided by the expertise of community members [27, 28]. This highlights the need to improve outreach, relationship-building, and close consultation with First Nations communities in developing provider support tools in the province.

### Limitations

This study is limited in the conclusions that can be drawn regarding the service’s utility to patients and providers, given that direct patient outcome variables were not collected and limited details surrounding the consultations were recorded in order to maintain privacy and create a smooth workflow for the providers staffing the line. Instead, survey responses from clinicians who called the support line were used to assess the program’s efficacy and usability, which may introduce response bias. This survey method was limited by a low response rate of 20.2%, though this rate is consistent with a body of research documenting low response rates in similar physician-targeted surveys [29, 30]. The study findings are limited by missing data, particularly for providers’ clinical settings and geographic locations, as some providers opted to maintain privacy around their practice. Due to a need to implement the service rapidly, the evaluation did not include a cost-evaluation component and therefore cannot speak to its economic feasibility as a care model. However, the cost of untreated substance use disorders on our healthcare system is high, and the prevention of significant referrals to acute care and/or specialty settings is notable. Our findings may not be generalizable to other settings that face similar treatment gaps, as each context is subject to its own health policies, clinical resources, and patient populations. Yet, the similarly positive results from provider support services in other nations suggest that this model may be applied flexibly to unique patient populations and geographies [16]. 

## Conclusions

In British Columbia, the impact of the 24/7 Addiction Medicine Clinician Support Line was significant and widespread, increasing accessibility to point-of-care and evidence-based addictions treatment across a wide range of care settings. The intervention is the first of its kind in North America, and its relatively simple model of virtual consultation is scalable and adaptable to other healthcare settings. The addiction medicine provider consultation model holds the potential to improve clinician support and increase crucial access to evidence-based care for patients with substance use disorders, particularly in under-resourced settings.

## Electronic supplementary material

Below is the link to the electronic supplementary material.

Supplementary Material 1

## Data Availability

The datasets used and/or analyzed during the current study are available from the corresponding author on reasonable request.

## Declarations

### Statement of adherence to preprint policy

This article has not been posted on any preprint servers.
