# Supplementary material for: Expanding access to addictions care: Implementation of a 24-hour healthcare provider support line in British Columbia, Canada
Source: Addict Sci Clin Pract. 2024 Oct 31;19:76. doi: 10.1186/s13722-024-00508-z (PMC11526689; doi:10.1186/s13722-024-00508-z)
Supplement: Supplementary file 1 — Supplementary Material 1 [file 13722_2024_508_MOESM1_ESM.docx]

ASCP Supplement

Supplement Table 1: Provider Post-Consultation Survey

| Post-Consult Provider Questionnaire  How satisfied were you with your call to the 24/7 Support Line? (yes/no)  Did this call avoid a patient referral to see a specialist? (yes/no)  Did this call avoid an emergency department visit? (yes/no)  Did this call shorten the time it would have otherwise taken for your patient to receive treatment? (yes/no)  Do you feel you were able to provide your patient with better care as a result of this call? (yes/no)  Was a prescription initiated as a result of the call? (yes/no)  Was a prescription changed as a result of the call? (yes/no) |
| --- |
